# Aging and Metabolism: Two Sides of the Same Coin

**DOI:** 10.1016/j.ebiom.2017.07.004

**Published:** 2017-07-08

Findings from the most recent Global Burden of Disease study, published in October 2016 in *The Lancet*, show that in 2015, the mean life expectancy worldwide had reached roughly 72 years. This is an increase of 10 years since 2000, largely attributed to improvements in childbirth-associated and infant mortality, better management of HIV/AIDS, and more efficient vaccine and drug delivery to combat other communicable diseases. These healthcare efficiencies mean that, for the first time in history, the average child born anywhere in the world today is expected to live to at least 60 years. By 2050, 2.1 billion people—roughly 25% of the global population—will be older than 60 years.

Yet further analyses of these epidemiological data suggest that over the same period between 2000 and 2015, which saw a 10 year increase in overall life expectancy, there was a much more modest increase in healthy life expectancy (HALE)—a measure that takes into account years lived with disability—of 2.9–3.5 years (for men and women, respectively). For those older than 65 years of age, HALE increased by just 0.85–1.2 years. It becomes clear that, despite tremendous medical advances contributing to extension of lifespan over recent decades, a concomitant increase in quality of life at older ages has not kept pace.

The mounting challenges healthcare systems face with an aging population are largely due to increased prevalence of noncommunicable diseases (NCDs). In 2015, NCDs accounted for 70% of all deaths globally. 80% of NCD-related deaths are attributed to cardiovascular disease, cancer, respiratory diseases, and diabetes. Indeed, data from the US Surveillance, Epidemiology, and End Results (SEER) Program suggest that incidence of these NCDs increases exponentially with every year lived beyond the age of 50 years. Despite mounting evidence that aging increases chronic disability and premature death, studies on healthy aging to tackle this problem are in their infancy. Not until May 2016, as part of its broader Sustainable Development Goals (SDG) to “ensure healthy lives and promote well-being for all at all ages”, did the UN formulate a strategy to address healthy aging. In its initial stage, to be completed by 2020, part of this strategy will be to establish a framework for what healthy aging entails, taking into account evidence-based metrics of quality of life and functional abilities in daily activities of older individuals. Additionally, on June 7, 2017, the UN Economic and Social Council adopted a resolution to increase resources towards and take greater action against NCDs that overwhelmingly affect aged patients.

In this issue of *EBioMedicine,* we dedicate a section focused on Aging and Metabolism, with a series of In Focus and Review articles discussing diverse aspects of geroscience—the relatively new field of understanding the biology of aging and age-related disease. At the core of geroscience research is the dogma that aging is not simply an immutable outcome of life, but that its biological underpinnings, once understood, can be manipulated to improve health. From the series of pieces presented in this issue, it becomes apparent that aging and age-related disease are intimately entangled with metabolic function, both at the molecular/cellular and organismal levels. The etiology of cardiovascular disease, cancer, lung, liver, and kidney dysfunction, and diabetes can be at least in part attributed to, with varying degrees, impairments in lipid handling and storage, immunometabolism, susceptibility to cellular stressors, glucose utilization and insulin sensitivity, and other metabolic defects associated with increasing age.

Cellular senescence describes the phenomenon where somatic cells cease to divide, become resistant to apoptosis, and develop a senescence-associated secretory phenotype (SASP) that can have deleterious effects on surrounding tissues and throughout the body. (Thomas von Zglinicki and colleagues) discuss the role of mitochondrial dysfunction in cellular senescence and how breakdown of mitochondrial components (mitophagy) is likely involved in senescence and aging. How telomeres—irrespective of length, contrary to the previous notion that shortened telomeres were simply a readout of a cell's age—can both protect against and effect cellular senescence programs is discussed by (João Passos and colleagues).

Translational approaches to targeting the biological basis of aging is a rapidly-developing field. (James Kirkland and colleagues) discuss targeting cellular senescence programs to improve fitness. Among these approaches are so-called senolytic agents, which selectively clear senescent cells and relieve the associated pathophysiology they confer. Another approach is caloric restriction. Recapitulated from yeast to invertebrate animals to rodents to primates including humans, stringently regulating food intake has shown beneficial effects on longevity and health. (Rozalyn Anderson and colleagues) discuss some of the underlying mechanisms of caloric restriction and how they may be harnessed to intervene in age-related phenotypes. (Nancy Nadon and colleagues) elaborate on dietary interventions as part of the US National Institute on Aging's Interventions Testing Program to identify modifiers of lifespan in pre-clinical models before translation to human trials.

Accurately defining one's age may assist with the timing of appropriate interventions. Instead of using chronological age and epidemiological data to assess a patient's risk of age-related function or illness, (Sara Hägg and colleagues) discuss predictors of biological age, utilizing molecular markers to reliably predict one's personalized risks. Finally, (Michael Wade and colleagues) discuss frailty, with particular attention to an individual's postural control as not only a readout of overall fitness but also as a key target for improving quality of life.

This special section on Aging and Metabolism highlights both the considerable impending need healthcare systems must meet in managing an aging population, but also the remarkable progress researchers have made and continue to make to meet these needs. We wish to thank the authors of the In Focus and Review articles presented in this issue of *EBioMedicine* for emphasizing the diverse issues being tackled by the geroscience community, as well as consultants Thomas von Zglinicki and Rozalyn Anderson who helped shape this special issue section, and the referees who provided valuable feedback to strengthen each manuscript. Given the enthusiastic commitment among the geroscience community to—as the UN SDG states—ensure healthy lives and promote well-being for all at all ages, *EBioMedicine* looks forward to the continued rapid development of this burgeoning field.

*EBioMedicine*

